# A case report of hemophagocytic lymphohistiocytosis occurring during ibrutinib chemotherapy with a literature review

**DOI:** 10.1097/MD.0000000000045957

**Published:** 2026-01-16

**Authors:** Xiaonan Hou, Manzhi Wang, Shengchao Miao, Yan Guo

**Affiliations:** a Department of Hematology, The First Affiliated Hospital of Shandong First Medical University, Shandong, China.

**Keywords:** case report, chronic lymphocytic leukemia, hemophagocytic lymphohistiocytosis, HLH-94 protocol, ibrutinib

## Abstract

**Rationale:**

Ibrutinib, a Bruton tyrosine kinase inhibitor, is widely used in chronic lymphocytic leukemia (CLL). Its association with secondary hemophagocytic lymphohistiocytosis (HLH) is exceedingly rare but potentially fatal.

**Patient concerns:**

A 59-year-old male with CLL presented with recurrent fever and progressive splenomegaly.

**Diagnoses:**

Bone marrow biopsy confirmed CLL. Five months after ibrutinib (420 mg/day) initiation, progressive pancytopenia, hyperferritinemia, and hemophagocytosis on repeat marrow aspirate established HLH diagnosis.

**Interventions:**

HLH-94 protocol (etoposide + dexamethasone) was initiated.

**Outcomes:**

The patient developed fungemia followed by massive cerebral infarction. Treatment was withdrawn due to deterioration, resulting in death.

**Lessons:**

Ibrutinib may trigger HLH via immune dysregulation. Early recognition through vigilant monitoring of cytopenias, ferritin, and marrow hemophagocytosis is critical to improve survival.

## 1. Introduction

Hemophagocytic syndrome, also known as hemophagocytic lymphohistiocytosis (HLH), is a syndrome characterized by the proliferation and activation of lymphocytes and macrophages induced by a cytokine storm, accompanied by the phagocytosis of blood cells. The clinical prognosis of this condition is extremely poor. The etiological factors of HLH are diverse. Among them, drug-induced HLH has a low incidence and is relatively rare, making its diagnosis difficult. Our hospital admitted a patient with chronic lymphocytic leukemia (CLL) who developed hemophagocytic syndrome after treatment with ibrutinib. We now report the diagnostic and therapeutic process of this case and review relevant literature.

## 2. Case presentation

The patient is a 59-year-old male who was admitted to our hospital’s Respiratory Intensive Care Unit on January 7, 2023, due to “fever, cough accompanied by chest tightness for 10 days.” Ten days prior to admission, the patient developed fever, reaching a maximum temperature of 39°C. The complete blood count (CBC) results showed: WBC 70.66 × 10^9^/L, neutrophils 16.26 × 10^9^/L, lymphocytes 52.01 × 10^9^/L, and hemoglobin 87 g/L. Chest DR revealed bilateral pneumonia, mediastinal lymphadenopathy, and splenomegaly. Physical examination revealed an anemic appearance. On lung auscultation, coarse breath sounds were noted bilaterally, accompanied by scattered moist rales. The diagnosis was “severe pneumonia and severe anemia.” The patient received antimicrobial therapy, and a bone marrow smear cytology examination was performed, which showed a significant increase in the proportion of lymphocytes, suggesting the possibility of B-cell lymphoproliferative disorder. Bone marrow biopsy showed the marrow is markedly hypercellular, with significant proliferation of small B lymphocytes between the bone trabeculae. The cells were positive for CD20 and CD23, consistent with chronic lymphocytic leukemia/small B-cell lymphoma. Flow cytometry indicated that B-cell lymphoma had invaded the bone marrow, with positive results for CD19, CD20, CD5, CD23, and Kappa, and negative results for CD103, CD10, FMC7, and Lambda. The patient was diagnosed with CLL. After aggressive anti-infectious and symptomatic treatment, the hemoglobin and platelet levels improved compared to before. The patient was followed up and observed. Subsequent regular abdominal ultrasound examinations revealed progressive splenomegaly in the patient, which met the treatment indications for CLL. Therefore, on March 27, 2023, the patient was administered ibrutinib at a dosage of 420 mg once daily. During ibrutinib treatment, with regular outpatient and inpatient follow-ups (Table 1), on August 26, 2023, after 5 months of administration, the patient experienced agranulocytosis, thrombocytopenia, and fever without any apparent precipitating factors. The CBC results showed: WBC 2.28 × 10^9^/L, neutrophils 0.09 × 10^9^/L, hemoglobin 122 g/L, and Plt 90 × 10^9^/L. Peripheral blood flow cytometry revealed 4.84% aberrant B lymphocytes. The cytopenia was attributed to hematologic toxicity of ibrutinib, prompting dose reduction to 140 mg/day. Supportive therapies including hematopoietic stimulation, leukopenia management, thrombocytopenia intervention, and anti-infective therapy were administered. The patient achieved normalization of leukocyte counts and remained afebrile. Following discharge, ibrutinib was resumed at the standard dose of 420 mg/day.

**Table 1:** Patient follow-up.

Date	WBC	N	L	Hb	Plt	Spleen
April 1, 2023	46.51	5.58	39.068	122	115	55 × 180
April 22, 2023	6.50	2.80	3.3	122	114	
May 20, 2023	4.51	2.66	1.45	111	147	59 × 203
June 24, 2023	5.01	1.73	2.90	120	149	
July 22, 2023	5.39	2.93	2.09	120	152	75 × 165
August 26, 2023	2.28	0.09	1.81	122	90	51 × 175
August 31, 2023	5.00	2.17	2.38	108	72	
October 2, 2023	2.11	0.90	1.06	90	52	

Hb = hemoglobin, L = lymphocytes, N = neutrophils.

**Figure 1.**
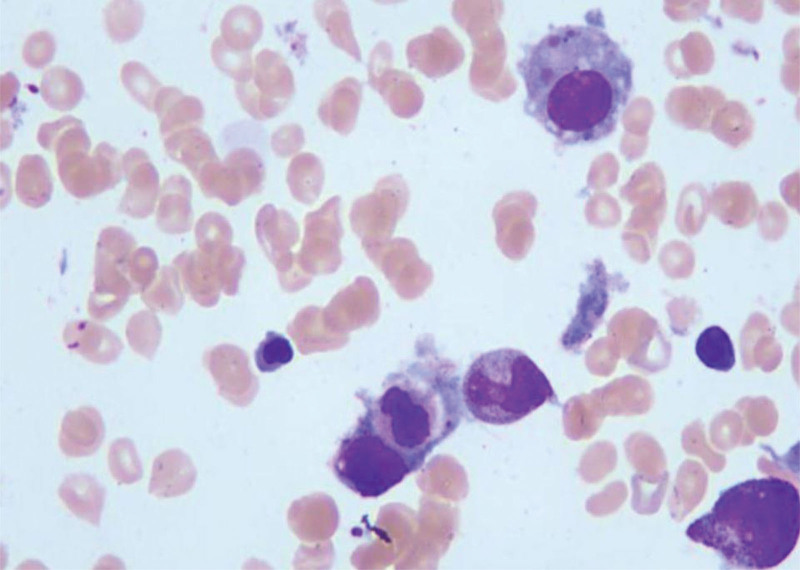
Bone marrow smear showing hemophagocytes in a CLL patient who developed hemophagocytic syndrome during ibrutinib therapy. Wright stain 1000×. CLL = chronic lymphocytic leukemia.

In October 2023, the patient developed recurrent fever. Follow-up CBC demonstrated progressive trilineage cytopenia (Table 1). Comprehensive infectious workup revealed unremarkable procalcitonin and C-reactive protein levels. Epstein–Barr virus (EBV) DNA, cytomegalovirus DNA, G-test, and GM-test were all negative. Chest imaging demonstrated no interval change in pulmonary lesions. Serial blood cultures remained sterile, with no clinical evidence of infection at other sites. Given the lack of response to empirical anti-infective therapy, infectious etiology was deemed unlikely. Additional workup revealed: the ferritin level was 3041.52 ng/mL, and the soluble CD25/IL-1Ra test result was 23000.52 pg/mL. Bone marrow smear cytology examination showed that post-CLL treatment, the proportion of erythroid cells was significantly increased, and hemophagocytic cells were visible (Fig. 1). According to the HLH-2004 criteria established by the Histiocyte Society,^[1]^ The patient presented with typical manifestations of hemophagocytic syndrome, including persistent fever, cytopenia, hemophagocytosis in the bone marrow, elevated serum ferritin levels, and increased soluble interleukin-2 receptor levels,confirming the diagnosis of HLH. Further evaluation for underlying triggers of hemophagocytosis revealed no evidence of primary disease progression. Although PET-CT was not performed due to financial constraints, bone marrow aspiration demonstrated hypercellularity with frequent hemophagocytic cells and characteristic smudge cells. Flow cytometry detected 8.64% abnormal small B lymphocytes (CD5+/CD23+/Kappa-restricted), consistent with the known CLL immunophenotype. Serial ultrasound monitoring showed progressive splenic reduction without new lymphadenopathy. Comprehensive assessment ruled out Richter transformation or other indicators of CLL progression, suggesting the hemophagocytic syndrome was more likely triggered by immune dysregulation or occult infection rather than malignancy evolution. Genetic evaluation: comprehensive HLH-associated genetic screening (56 genes including AK2, AP3B1, CD27, CD70, UNC13D, and TP53) identified no clinically significant mutations, small insertions, or deletions within the analyzed regions. Given the exclusion of CLL progression, primary HLH, and active infection, drug-induced HLH was strongly suspected. The patient was receiving ibrutinib therapy, and prior case reports have documented ibrutinib-associated HLH. A strict temporal correlation between ibrutinib exposure and HLH onset (initiated 6 months prior) further supported this etiology. While definitive causality requires rechallenge verification (deemed unethical here), ibrutinib was considered a plausible culprit based on the Naranjo adverse drug reaction probability scale (score ≥ 5).

Following the diagnosis of HLH, ibrutinib was discontinued and the HLH-94 protocol (dexamethasone + etoposide) was initiated. During the bone marrow suppression phase following chemotherapy, the patient developed fungalemia. Next-generation sequencing analysis of the bloodstream revealed infections with *Rhizopus* and *Aspergillus*. Subsequently, extensive cerebral infarction occurred, strongly suggested to result from septic emboli due to hematogenous dissemination of mucormycosis. Deep fungal infection is an opportunistic infection and invasive process in critically ill patients due to immune deficiency.^[2]^ Chronic use of immunosuppressants and chemotherapy are both risk factors for fungal infections.^[3]^ As a type of fungus, *Mucormycetes* exhibit extremely strong invasiveness, with their hyphae capable of invading blood vessels and causing hematogenous dissemination. The growth of hyphae within the vascular lumen leads to endothelial damage and vessel wall destruction, subsequently resulting in thrombosis and lumen occlusion.^[4]^ When these hyphae invade cerebral arteries, they can lead to cerebral hemorrhage and cerebral infarction. We administered amphotericin B cholesterol sulfate for antifungal treatment. However, due to the patient’s critical condition and dismal prognosis, thorough goals-of-care discussions were held with the family, they chose to withdraw treatment and requested discharge with hospice care. During subsequent follow-up, it was confirmed that the patient had passed away.

## 3. Discussion

HLH is a life-threatening hematologic disorder with a dismal prognosis (median survival < 2 months if untreated). It is characterized by dysfunctional T-cell and natural killer (NK)-cell responses, triggering a cytokine storm, disseminated intravascular coagulation, multi-organ failure, and high mortality.^[5]^ Based on the presence or absence of related immune deficiencies, HLH is classified into primary HLH and secondary HLH forms. Primary HLH is mostly observed in children under the age of 2 and is primarily caused by autosomal or X-linked recessive inheritance. Secondary HLH is more common in adults, and its immune dysfunction is characterized by reversible NK or CD8 + T-cell dysfunction. It often occurs secondary to infections caused by viruses, bacteria, parasites, etc, as well as rheumatic/autoimmune diseases, medications, tumors, metabolic-related diseases, and other conditions.^[6]^ Among these, infection is the most common etiology,^[7]^ especially EBV infection. Or NK cell deficiency, which frequently observed during chemotherapy or in the setting of sepsis.^[8,9]^ Among malignancies, lymphoma represents the most prevalent hematologic neoplasm. According to current literature and expert consensus.^[10]^ Based on the temporal relationship to lymphoma diagnosis and treatment, HLH is classified into 2 distinct entities: lymphoma-triggered HLH, which may occur prior to formal diagnosis, at initial diagnosis, or during disease progression/relapse; and treatment-emergent HLH, which develops specifically during cytotoxic chemotherapy for lymphoma management. Based on the etiology, it is further classified into the following 3 types, including HLH directly induced by lymphoma. This primarily occurs because cytokines (such as interleukin-6, interferon-gamma, etc) secreted by lymphoma lead to a hyperinflammatory state, which may contribute to the development of HLH. And there is also HLH caused by infections and immunotherapy. The former occurs when, during the treatment of lymphoma, the patient’s immune function is suppressed, making them susceptible to various bacterial, fungal, and viral infections, which can lead to HLH. The latter primarily occurs after immunotherapy such as chimeric antigen receptor T-cell therapy or immune checkpoint inhibitors, and in rare cases, can progress to fulminant HLH.^[11,12]^

The patient’s medical history has ruled out common causes such as CLL progression, infection, and congenital genetic abnormalities. We speculate that HLH was likely caused by ibrutinib, although there is currently limited literature reporting cases of drug-induced HLH.^[13]^ Ibrutinib, as a targeted agent, is a covalent inhibitor of Bruton tyrosine kinase (BTK). It exerts anti-tumor effects by inhibiting BTK and downstream signaling pathways and has been a 1st-line treatment for CLL. However, ibrutinib does not selectively inhibit BTK and exhibits off-target effects, potentially inhibiting other kinases such as interleukin-2-inducible T-cell kinase (ITK), leading to impaired NK cell degranulation,^[14]^ ITK is a critical molecule in the T-cell receptor signaling pathway, particularly within Th2 and CD8 + T cells. Inhibition of ITK may bias the immune response toward promoting Th1 and cytotoxic T-cell responses. This shift toward a Th1-type response can lead to the robust production of pro-inflammatory cytokines, such as interferon-gamma.^[15]^ Elevated levels of interferon-gamma are one of the most potent triggers for activating macrophages and monocytes. These hyperactivated macrophages, in turn, robustly produce other inflammatory cytokines, such as tumor necrosis factor-alpha, interleukin-6, interleukin-1β, and interleukin-18. Activated T cells and macrophages mutually stimulate each other, forming a positive feedback loop that ultimately leads to uncontrolled, “storm-level” release of cytokines, thereby inducing hemophagocytic syndrome.^[13]^ Second-generation BTK inhibitors such as zanubrutinib and acalabrutinib can reduce off-target effects by improving target selectivity. Side effects like atrial fibrillation and bleeding are significantly reduced compared to ibrutinib. Moreover, no literature reports have currently documented HLH associated with BTK inhibitors other than ibrutinib.

In addition to the pharmacological mechanisms of ibrutinib itself that may trigger HLH, certain underlying patient-specific factors can also contribute to the development of this syndrome. Some patients may carry heterozygous mutations or specific polymorphisms in genes such as PRF1, UNC13D, STX11, and STXBP2, which encode perforin, Munc13-4, syntaxin-11, and Munc18-2 proteins, respectively.^[16]^ These proteins play critical roles in the cytotoxic function of NK cells and cytotoxic T lymphocytes. Impairment in their expression or function may lead to compromised activity of NK cells and cytotoxic T lymphocytes.^[17]^ Thereby increasing the risk of HLH. Moreover, EBV infection is the most common infectious trigger of HLH. Ibrutinib treatment can cause immunosuppression, potentially leading to EBV reactivation, which may induce a cytokine storm and promote the development of HLH. It is also worth noting that some patients who have previously undergone multiple lines of chemotherapy or potent immunosuppressive therapies may already have severely impaired or dysregulated T-cell function. The addition of ibrutinib in such cases could further disrupt immune balance, significantly increasing the likelihood of HLH occurrence. In this study, potential triggers for HLH mentioned above have been excluded in this patient.

HLH induced by ibrutinib is rare, and there have been very few relevant case reports to date.^[18]^ In 2016, Poole et al^[19]^ reported a case of a CLL patient who developed HLH following ibrutinib administration, leading to rapid multi-organ failure and subsequent death. This case illustrates the pathophysiological mechanisms by which ibrutinib’s impact on the immune system resulted in the occurrence of HLH. Thus, further research is warranted to investigate the correlation between ibrutinib and the development of HLH. Despite timely diagnosis and treatment, the patient’s condition progressed rapidly and was accompanied by multiple complications, ultimately leading to death.

## 4. Conclusion

From the diagnosis and treatment of this patient, we have gained the following insights: In the context of CLL treatment, if ibrutinib-associated HLH is suspected or confirmed, ibrutinib should be discontinued immediately, and a thorough investigation of potential triggers for HLH must be conducted, with emphasis on PCR testing for viruses such as EBV and cytomegalovirus. If active viral infection is detected, appropriate antiviral therapy should be initiated. Given the immunocompromised state of patients due to both the underlying disease and long-term BTK inhibitor therapy, adherence to the HLH-94 (dexamethasone + etoposide) or HLH-2004 protocols may increase the risk of severe infections. Alternative approaches, such as novel targeted agents including Janus Kinase inhibitors, could be considered, though the optimal treatment strategy requires further exploration. However, treatment should adhere to the principle of individualization, with early diagnosis and early treatment to prevent and manage complications, thereby improving patient prognosis and increasing survival rates.

## Author contributions

**Conceptualization:** Yan Guo.

**Data curation:** Xiaonan Hou, Shengchao Miao, Yan Guo.

**Investigation:** Xiaonan Hou, Manzhi Wang.

**Writing – original draft:** Xiaonan Hou.

**Writing – review & editing:** Yan Guo.

## References

[1] Henter JI Horne A Aricó M. HLH-2004: diagnostic and therapeutic guidelines for hemophagocytic lymphohistiocytosis. Pediatr Blood Cancer. 2007;48:124–31. 16937360 10.1002/pbc.21039

[2] Singhi S Deep A. Invasive candidiasis in pediatric intensive care units. Indian J Pediatr. 2009;76:1033–44. 19907936 10.1007/s12098-009-0219-6

[3] Wang YW Jing LJ Yao YB. Risk factors analysis for deep fungal infection in patients with large area cerebral infarction [Chinese]. Zhongguo Shi Yong Shen Jing Ji Bing Za Zhi. 2016;19:39–41.

[4] Palacios E Rojas R Rodulfa J González-Toledo E. Magnetic resonance imaging in fungal infections of the brain. Top Magn Reson Imaging. 2014;23:199–212. 24887690 10.1097/RMR.0000000000000025

[5] Cavallari M Ciccone M Falzoni S. Hemophagocytic lymphohistiocytosis after EBV reactivation and ibrutinib treatment in relapsed/refractory chronic lymphocytic leukemia. Leuk Res Rep. 2017;7:11–3. 28180067 10.1016/j.lrr.2017.01.002 PMC5288319

[6] Ramos-Casals M Brito-Zerón P López-Guillermo A Khamashta MA Bosch X. Adult haemophagocytic syndrome. Lancet. 2014;383:1503–16. 24290661 10.1016/S0140-6736(13)61048-X

[7] Lee WI Chen SH Hung IJ. Clinical aspects, immunologic assessment, and genetic analysis in Taiwanese children with hemophagocytic lymphohistiocytosis. Pediatr Infect Dis J. 2009;28:30–4. 19057461 10.1097/INF.0b013e3181813592

[8] Brisse E Wouters CH Matthys P. Advances in the pathogenesis of primary and secondary haemophagocytic lymphohistiocytosis: differences and similarities. Br J Haematol. 2016;174:203–17. 27264204 10.1111/bjh.14147

[9] Andaluz-Ojeda D Iglesias V Bobillo F. Early natural killer cell counts in blood predict mortality in severe sepsis. Crit Care. 2011;15:R243. 22018048 10.1186/cc10501 PMC3334794

[10] Wei N Wang Z. Interpretation of Chinese expert consensus recommendations for the diagnosis and management of hemophagocytic lymphohistiocytosis associated with lymphoma (2022) [Chinese]. Zhonghua Yi Xue Za Zhi. 2022;102:1779–81. 35725360 10.3760/cma.j.cn112137-20220208-00252

[11] Brudno JN Kochenderfer JN. Recent advances in CAR T-cell toxicity: mechanisms, manifestations and management. Blood Rev. 2019;34:45–55. 30528964 10.1016/j.blre.2018.11.002 PMC6628697

[12] Frey N Porter D. Cytokine release syndrome with chimeric antigen receptor T cell therapy. Biol Blood Marrow Transplant. 2019;25:e123–7. 30586620 10.1016/j.bbmt.2018.12.756

[13] Teachey DT Rheingold SR Maude SL. Cytokine release syndrome after blinatumomab treatment related to abnormal macrophage activation and ameliorated with cytokine-directed therapy. Blood. 2013;121:5154–7. 23678006 10.1182/blood-2013-02-485623 PMC4123427

[14] Kohrt HE Sagiv-Barfi I Rafiq S. Ibrutinib antagonizes rituximab-dependent NK cell-mediated cytotoxicity. Blood. 2014;123:1957–60. 24652965 10.1182/blood-2014-01-547869 PMC3962169

[15] Dubovsky JA Beckwith KA Natarajan G. Ibrutinib is an irreversible molecular inhibitor of ITK driving a Th1-selective pressure in T lymphocytes. Blood. 2013;122:2539–49. 23886836 10.1182/blood-2013-06-507947 PMC3795457

[16] Gu J An N Wang X Xiao M Luo H. UNC13D c.2588G>A nucleotide variant impairs NK-cell cytotoxicity in adult-onset EBV-associated hemophagocytic lymphohistiocytosis: a pedigree study. Int J Mol Sci . 2025;26:8683. 40943599 10.3390/ijms26178683 PMC12429805

[17] Bryceson YT Pende D Maul-Pavicic A. A prospective evaluation of degranulation assays in the rapid diagnosis of familial hemophagocytic syndromes. Blood. 2012;119:2754–63. 22294731 10.1182/blood-2011-08-374199

[18] Ambinder AJ Hambley B Shanbhag S Merrill SA. Ibrutinib-associated hemophagocytic lymphohistiocytosis: a case series from Johns Hopkins. Am J Hematol. 2019;94:E296–9. 31400009 10.1002/ajh.25611

[19] Poole A Girard N Clayton F Tantravahi SK. Rapid onset of hemophagocytic lymphohistiocytosis in a patient with refractory chronic lymphocytic leukemia treated with ibrutinib. Leuk Lymphoma. 2017;58:1258–61. 27750463 10.1080/10428194.2016.1243679

